# Supplementary material for: Mutation characteristics and molecular evolution of ovarian metastasis from gastric cancer and potential biomarkers for paclitaxel treatment
Source: Nat Commun. 2024 May 4;15:3771. doi: 10.1038/s41467-024-48144-0 (PMC11069556; doi:10.1038/s41467-024-48144-0)
Supplement: Supplementary file 1 — Supplementary information [file 41467_2024_48144_MOESM1_ESM.pdf]

Supplementary Information

Mutation characteristics and molecular evolution of ovarian metastasis from gastric cancer and potential biomarkers for paclitaxel treatment

Pengfei Yu[1, *], Can Hu[1, *], Guangyu Ding[1, *], Xiaoliang Shi[2], Jingli Xu[1], Yang Cao[1], Xiangliu Chen[1], Wei Wu[3], Qi Xu[4], Jingquan Fang[1], Xingmao Huang[1], Shaohua Yuan[2], Hui Chen[2], Zhizheng Wang[2], Ling Huang[1], Fei Pang[2], Yian Du[1], Xiangdong Cheng[1, #]

[1]Department of Gastric Surgery, Zhejiang Cancer Hospital, Hangzhou Institute of Medicine (HIM), Chinese Academy of Sciences, Hangzhou, Zhejiang 310022, China.
[2]Shanghai OrigiMed Co., Ltd, Shanghai, 201114, P.R. China.
[3]Department of Pathology, Zhejiang Cancer Hospital, Hangzhou Institute of Medicine (HIM), Chinese Academy of Sciences, Hangzhou, Zhejiang 310022, China.
[4]Department of Oncology, Zhejiang Cancer Hospital, Hangzhou Institute of Medicine (HIM), Chinese Academy of Sciences, Hangzhou, Zhejiang 310022, China.
[*]These authors contributed equally: Pengfei Yu, Can Hu and Guangyu Ding
[#]Correspondence: Xiangdong Cheng. E-mail: abdsurg@163.com

Supplementary Figures

Supplementary Figure 1:

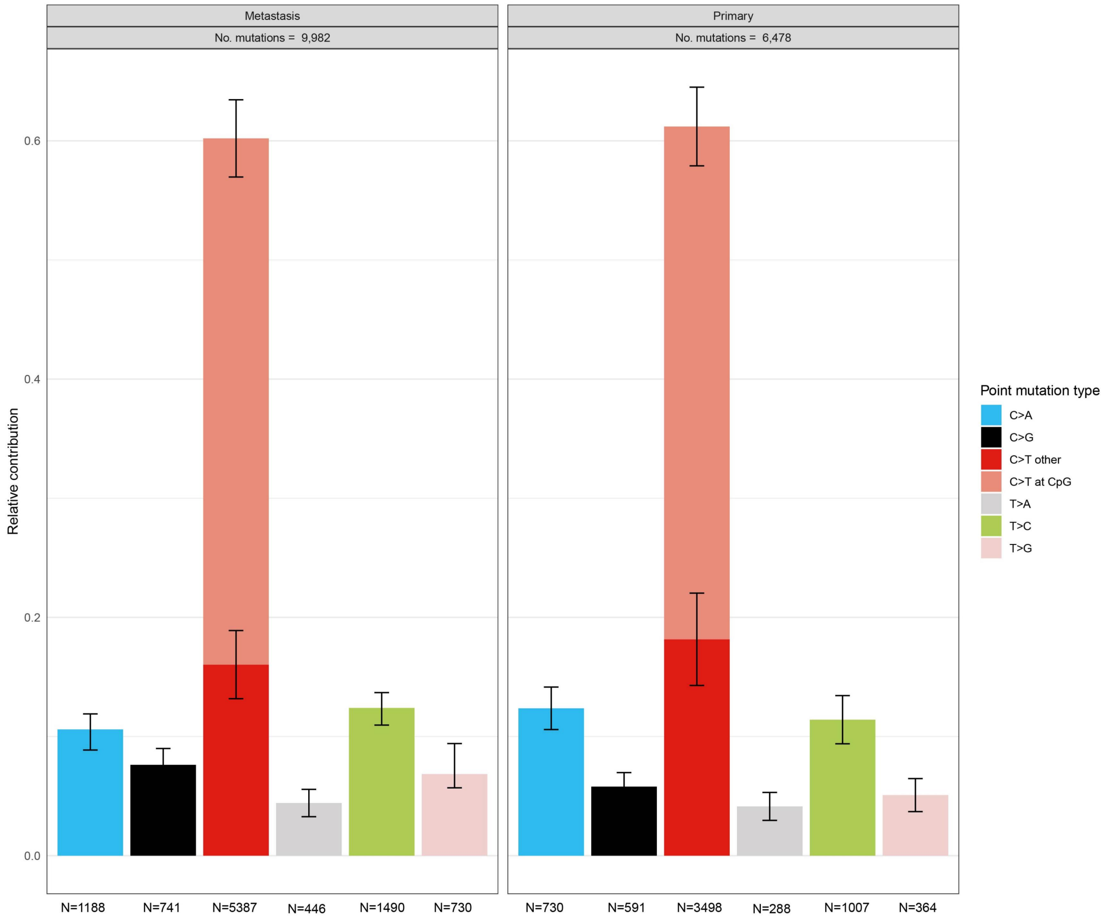

Distribution of the mutational signature in primary GC and metastatic ovarian samples.

Supplementary Figure 2:

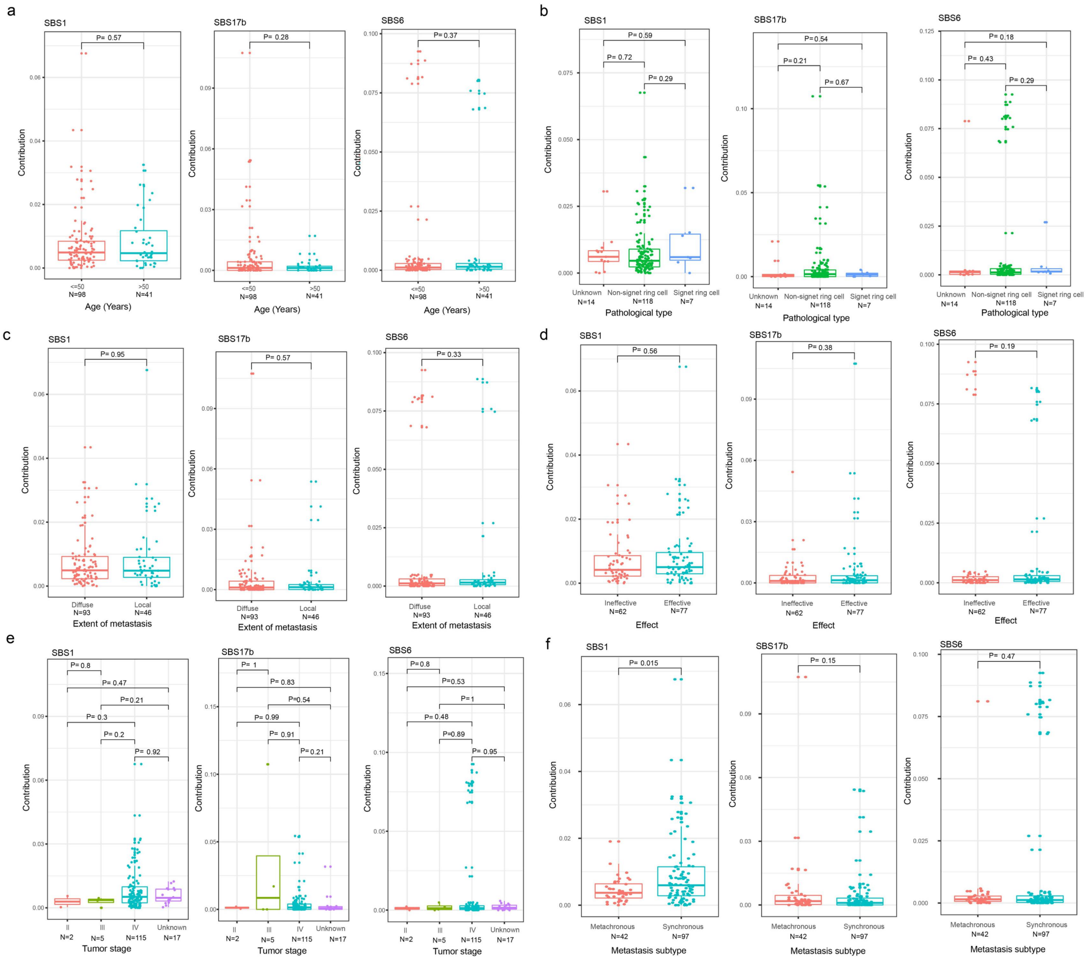

Correlation analysis between signatures and clinicopathological features such as age of patients a, histology type b, metastasis location c, efficacy of paclitaxel treatment d, tumor stage e, and metastasis subtype f. P-values were calculated using a one-way ANOVA analysis of variance with two-side. Source data are provided as a Source Data file.

Supplementary Figure 3:

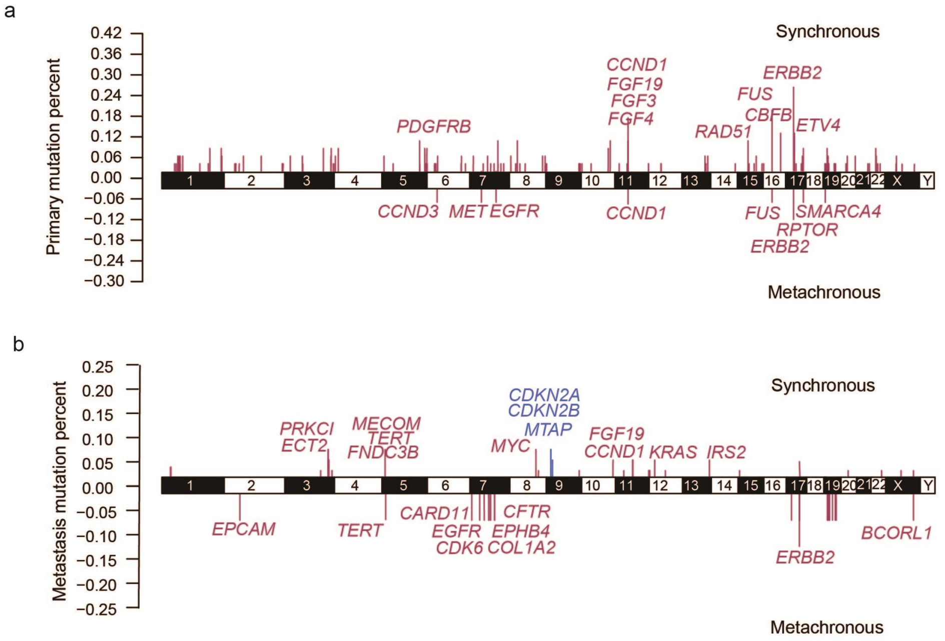

Comparison of chromosome distribution of CNVs between synchronous and metachronous ovarian metastasis in primary (a) and metastatic ovarian (b) lesions. a, the upper half (primary) represents the CNV distribution in synchronous primary gastric lesions. The lower half (primary) represents the CNV distribution in metachronous primary gastric lesions. b, the upper half (metastasis) represents the CNV distribution in synchronous ovarian metastasis tumors. The lower half (metastasis) represents the CNV distribution in metachronous ovarian metastasis tumors.

Supplementary Figure 4:

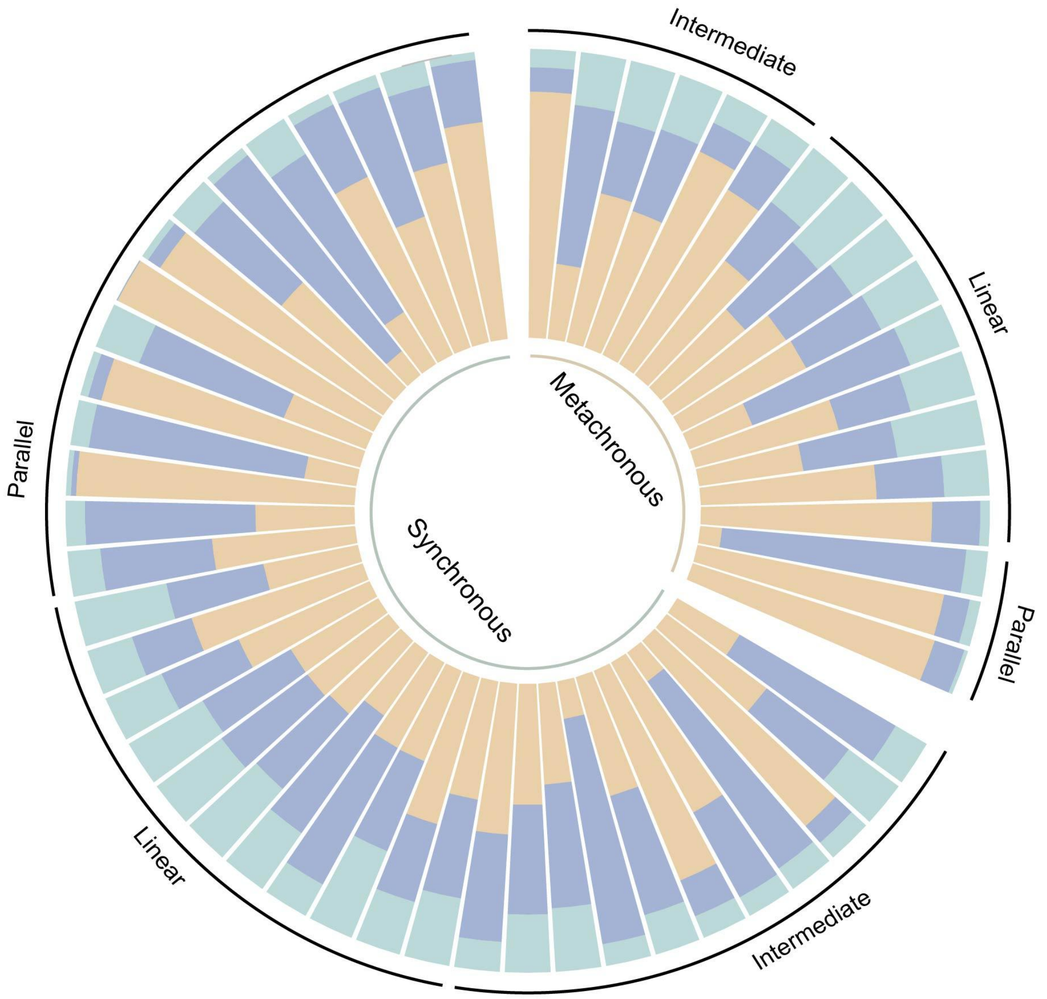

The distribution of three evolutionary groups of linear, intermediate and parallel in patients with synchronous or metachronous ovarian metastasis

Supplementary Figure 5:

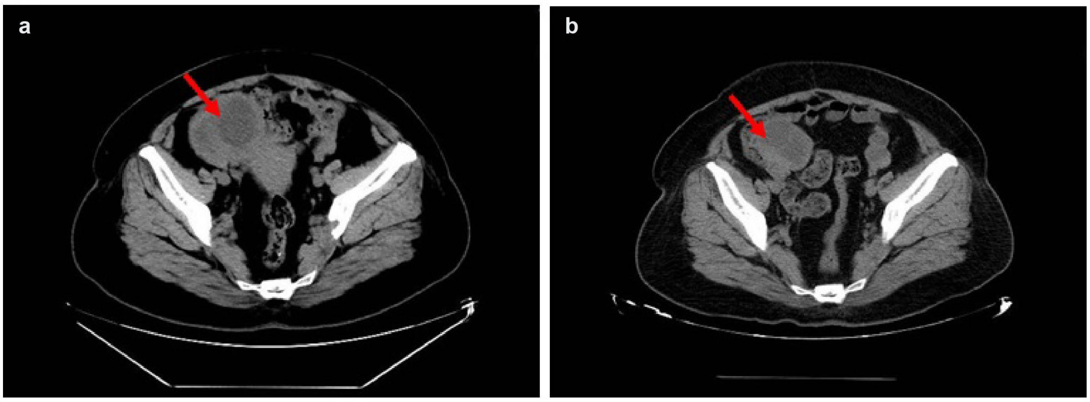

CT scan of ovarian metastasis from GC responding to paclitaxel. a Metastatic ovarian lesion from GC before treatment; b Significantly reduced metastatic ovarian lesion after 4 cycles of paclitaxel-based chemotherapy.

Supplementary Figure 6:

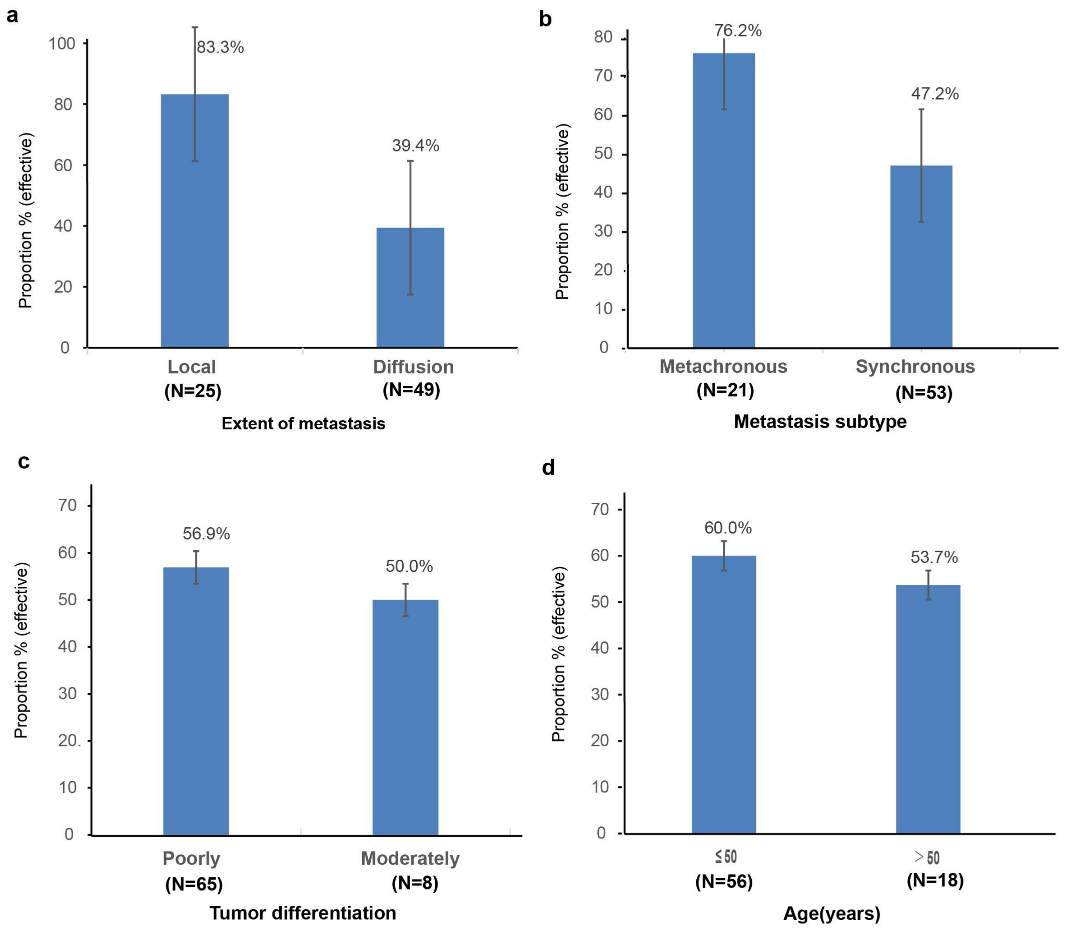

Correlation analysis between paclitaxel efficacy and metastasis loci (a), metastasis subtype (b), tumor differentiation (c), and age of patients (d).

Supplementary Figure 7:

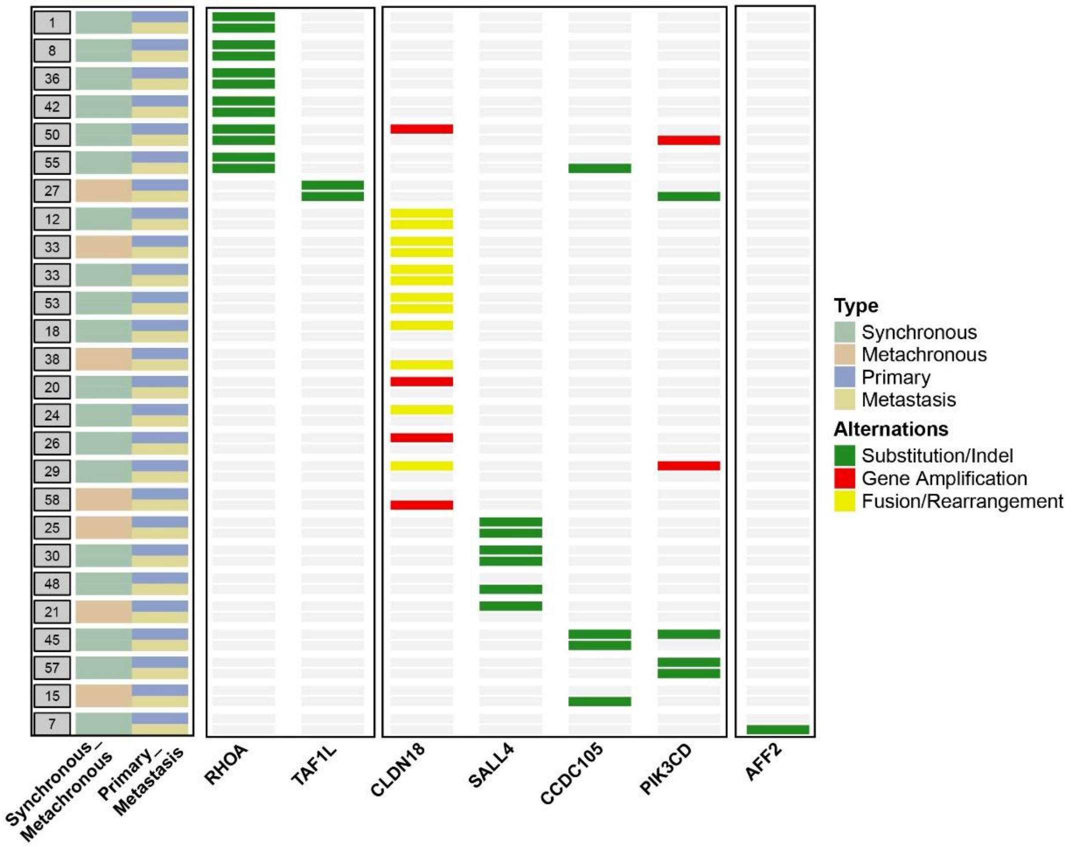

The distribution of seven paclitaxel efficacy related genes and other high-frequency genes in 64 paired samples. The upper part of each patient represents the mutation status of genes in the primary tumor. The lower part of each patient represents the mutation status of genes in ovarian metastatic tumors.

Supplementary Figure 8:

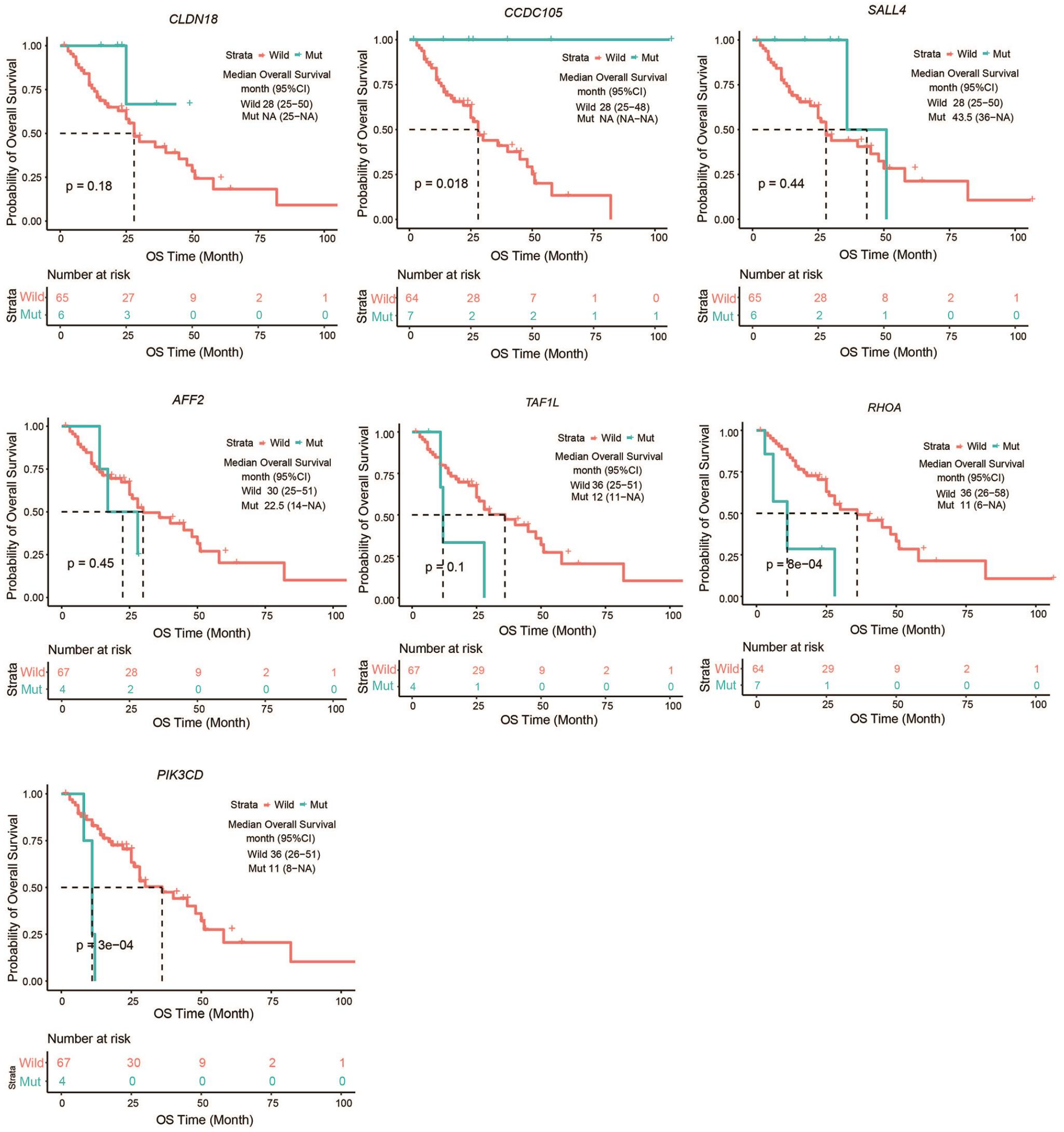

Associations between potential biomarkers related to paclitaxel efficacy such as CCDC105, CLDN18, SALL4, AFF2, PIK3CD, RHOA, and TAF1L and overall survival of patients. Differences between groups were assessed by the log-rank test. A p value < 0.05 was considered statistically significant.

Supplementary Figure 9:

a

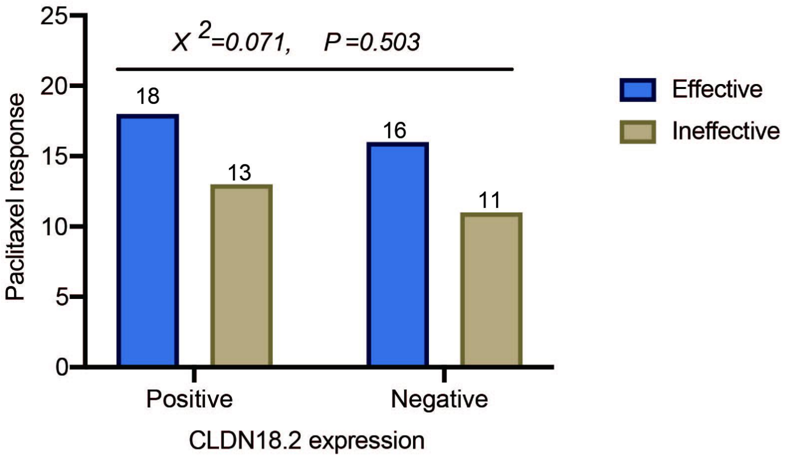

b

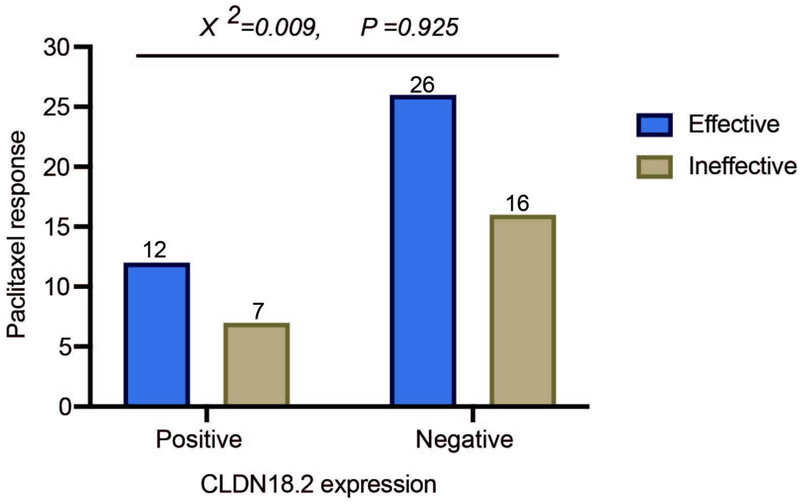

The correlation between CLDN18 protein expression and paclitaxel sensitivity in primary (a) or metastatic (b) lesions. Chi-squared test (χ^2) and Fisher's exact test were used in the comparison.

Supplementary Figure 10:

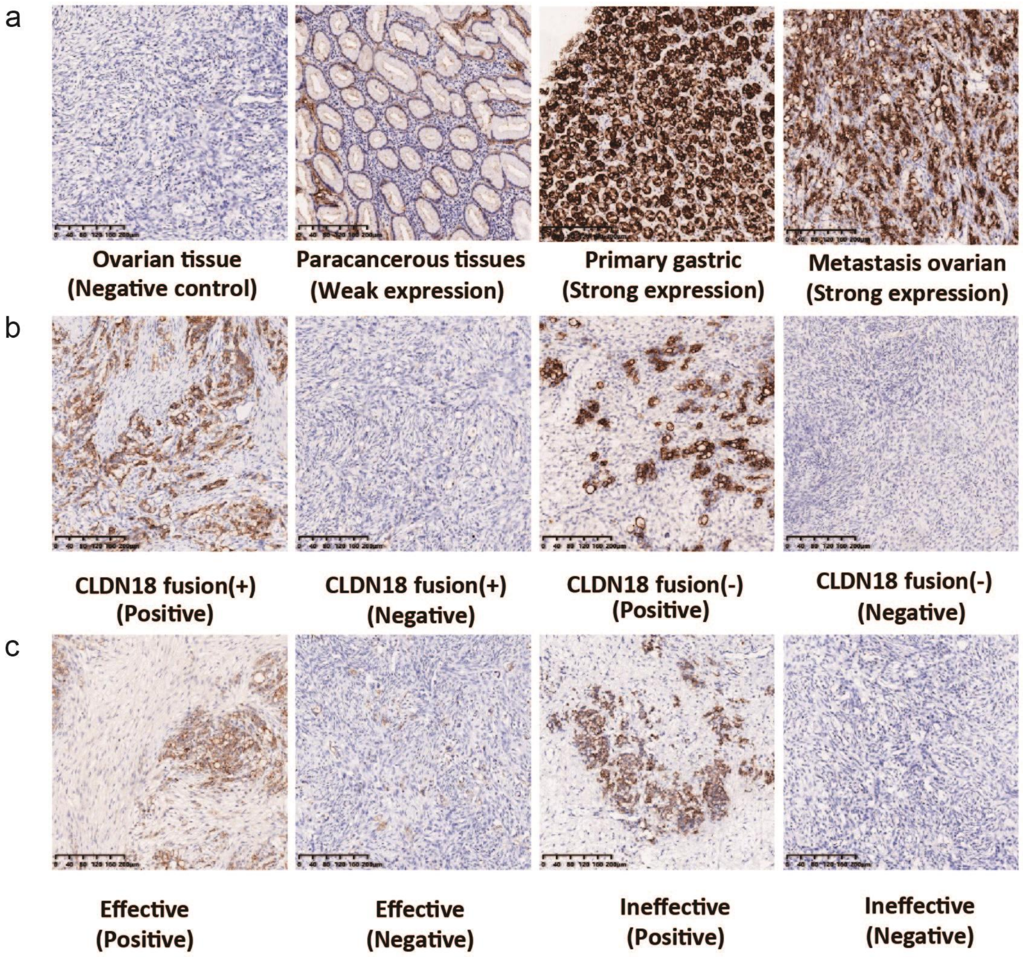

Correlation between CLDN18 protein expression and CLDN18 fusion. a Negative, weak, and strong expression of CLDN18 in patients with ovarian metastasis from GC. b The expression of CLDN18 is not associated with CLDN18 fusion. c The expression of CLDN18 is not correlated with the efficacy of paclitaxel.
