# Supplementary material for: Mutation characteristics and molecular evolution of ovarian metastasis from gastric cancer and potential biomarkers for paclitaxel treatment
Source: Nat Commun. 2024 May 4;15:3771. doi: 10.1038/s41467-024-48144-0 (PMC11069556; doi:10.1038/s41467-024-48144-0)
Supplement: Supplementary file 3 — Description of Additional Supplementary Files [file 41467_2024_48144_MOESM3_ESM.pdf]

Description of Additional Supplementary Files

Supplementary Data 1

Description: The similarity between the mutation features of our GC cohort and various known mutation features.

Supplementary Data 2

Description: Enriched pathways of primary gastric lesion, metastatic ovarian lesion, synchronous ovarian metastasis, metachronous ovarian metastasis and GC samples of TGGA database. Two-side P-values were calculated using Fisher’s exact test.

Supplementary Data 3

Description: The distribution of individual genomic alterations in primary gastric lesion and metastasis ovarian lesion.

Supplementary Data 4

Description: IHC detection of CLDN 18 in GC patients with ovarian metastasis.
