# Supplementary material for: Mutation characteristics and molecular evolution of ovarian metastasis from gastric cancer and potential biomarkers for paclitaxel treatment
Source: Nat Commun. 2024 May 4;15:3771. doi: 10.1038/s41467-024-48144-0 (PMC11069556; doi:10.1038/s41467-024-48144-0)
Supplement: Supplementary file 5 — Reporting Summary [file 41467_2024_48144_MOESM5_ESM.pdf]

nature portfolio

Corresponding author(s): Xiangdong Cheng

Last updated by author(s): Feb 26, 2024

Reporting Summary

Nature Portfolio wishes to improve the reproducibility of the work that we publish. This form provides structure for consistency and transparency in reporting. For further information on Nature Portfolio policies, see our Editorial Policies and the Editorial Policy Checklist.

Statistics

For all statistical analyses, confirm that the following items are present in the figure legend, table legend, main text, or Methods section.

n/a	Confirmed	
☐	☒	The exact sample size (*n*) for each experimental group/condition, given as a discrete number and unit of measurement
☐	☒	A statement on whether measurements were taken from distinct samples or whether the same sample was measured repeatedly
☐	☒	The statistical test(s) used AND whether they are one- or two-sided<br>*Only common tests should be described solely by name; describe more complex techniques in the Methods section.*
☒	☐	A description of all covariates tested
☐	☒	A description of any assumptions or corrections, such as tests of normality and adjustment for multiple comparisons
☐	☒	A full description of the statistical parameters including central tendency (e.g. means) or other basic estimates (e.g. regression coefficient) AND variation (e.g. standard deviation) or associated estimates of uncertainty (e.g. confidence intervals)
☐	☒	For null hypothesis testing, the test statistic (e.g. *F*, *t*, *r*) with confidence intervals, effect sizes, degrees of freedom and *P* value noted<br>*Give P values as exact values whenever suitable.*
☒	☐	For Bayesian analysis, information on the choice of priors and Markov chain Monte Carlo settings
☒	☐	For hierarchical and complex designs, identification of the appropriate level for tests and full reporting of outcomes
☒	☐	Estimates of effect sizes (e.g. Cohen's *d*, Pearson's *r*), indicating how they were calculated

Our web collection on statistics for biologists contains articles on many of the points above.

Software and code

Policy information about availability of computer code

Data collection

Tumor tissue samples and matched blood samples were collected for the determination of genomic alterations (GAs). Genomic DNA was isolated using a QIAamp DNA FFPE Tissue Kit and a QIAamp DNA Blood Midi Kit (Qiagen, Hilden, Germany), according to the manufacturer's instructions. The concentration of DNA was measured using Qubit and normalized to 20–50 ng/µL. WES libraries were prepared and captured using the SureSelect Human All Exon V6 kit (Agilent Technologies), according to the manufacturer's instructions, and then sequenced using an Illumina HiSeq X Ten system (Illumina, Inc., CA). WES was conducted with a mean coverage depth of 187x (range: 108-344x) for tumor samples, consistent with recommendations.

Data analysis

SNVs were identified using MuTect (v1.17). Insertions/deletions (Indels) were identified using PINDEL (V0.2.4). The functional impact of these mutations was annotated using SnpEff3.0. CNVs were identified using Control-FREEC (v9.4), with the following parameters: window = 50,000 and step = 10,000. Gene fusions were detected using an in-house pipeline. Gene rearrangements were assessed with the Integrative Genomics Viewer.
According to the number of different types of point mutations such as C > A/G > T, C > G/G > C, C > T/G > A, T > A/A > T, T > C/A > G, and T > G/A > C, a cluster analysis was performed in order to observe similarity in tumor samples. Extracted mutational features were compared with the pan-cancer catalogue for 94 known features cited in the cancer somatic mutation catalogue (COSMIC) database (https://cancer.sanger.ac.uk/signatures/) using Mutational Patterns packages (3.6.0). The similarity of mutational features was assessed based on a cosine similarity > 0.85, which indicated common features.
For each designated group—primary, metastasis, synchronous, metachronous, and TCGA cohorts—we ascertained the top 100 genes that demonstrated the highest mutation frequencies. These gene cohorts were then subjected to pathway enrichment analysis utilizing the Kyoto Encyclopedia of Genes and Genomes (KEGG) database to elucidate the significant biological pathways implicated in each group. Gene set clustering into corresponding KEGG pathways was executed via the DAVID (Database for Annotation, Visualization and Integrated Discovery) Bioinformatics Resources 6.8 platform. Pathways with FDR < 0.05 were defined as significantly enriched in GC.

All SNVs were used to construct phylogenetic tree based on the Lineage Inference for Cancer Heterogeneity and Evolution (LICHeE) method. Relying on the phylogeny model, LICHeE utilized the somatic SNV patterns of samples and their VAFs as lineage markers for reconstructing a phylogenetic tree. The genetic distance between all pairs of samples in each patient were calculated using Treeomics. Treeomics v1.9.0 were used with the default settings to reconstruct the phylogenies of the metastatic tumor using high-quality somatic variants and the CN alterations were identified by WES, respectively.

For manuscripts utilizing custom algorithms or software that are central to the research but not yet described in published literature, software must be made available to editors and reviewers. We strongly encourage code deposition in a community repository (e.g. GitHub). See the Nature Portfolio guidelines for submitting code & software for further information.

Data

Policy information about availability of data

All manuscripts must include a data availability statement. This statement should provide the following information, where applicable:

- Accession codes, unique identifiers, or web links for publicly available datasets
- A description of any restrictions on data availability
- For clinical datasets or third party data, please ensure that the statement adheres to our policy

[illegible]

Research involving human participants, their data, or biological material

Policy information about studies with human participants or human data. See also policy information about sex, gender (identity/presentation), and sexual orientation and race, ethnicity and racism.

Reporting on sex and gender	A total of 74 GC patients are female.
Reporting on race, ethnicity, or other socially relevant groupings	Reporting on the ethnic background of Chinese female gastric cancer patients, the majority of the study population consisted of individuals from the Han ethnic group.
Population characteristics	A total of 74 GC patients with ovarian metastasis including 53 synchronous and 21 metachronous were enrolled. The median age of these patients was 46 (range: 28-73) years.
Recruitment	From January 1, 2012 to December 31, 2020, 192 patients with ovarian metastasis were screened from 15,315 GC patients. After excluding 101 patients who did not receive ovarian resection or biopsy and 17 patients with insufficient tumor volume, 74 patients were finally enrolled for this study. No other features were used to screen the patient population.
Ethics oversight	The study of human tumor samples was performed according to the declaration of Helsinki and Good Clinical Practice and approved by the Ethical Committee of Zhejiang Cancer Hospital (IRB-2022-279). Informed written consent was obtained from all participants.

Note that full information on the approval of the study protocol must also be provided in the manuscript.

Field-specific reporting

Please select the one below that is the best fit for your research. If you are not sure, read the appropriate sections before making your selection.

☒ Life sciences ☐ Behavioural & social sciences ☐ Ecological, evolutionary & environmental sciences

For a reference copy of the document with all sections, see nature.com/documents/nr-reporting-summary-flat.pdf

Life sciences study design

All studies must disclose on these points even when the disclosure is negative.

Sample size	No sample size calculated in this study. 192 patients with ovarian metastasis were screened from 15,315 GC patients. A total of 74 GC patients with ovarian metastasis including 53 synchronous and 21 metachronous were enrolled. Considering the challenges associated with procuring primary and corresponding metastatic lesions from the same individual, the cohort comprising 74 patients who possess samples of both primary and ovarian metastatic lesions currently represents the most extensive known patient population diagnosed with GC with ovarian metastasis.
Data exclusions	101 patients who did not receive ovarian resection or biopsy and 17 patients with insufficient tumor volume.
Replication	All the NGS data were from patients, and no technical replication was done for each sample. The numble of replications is always mentioned in text, methods and figure legends. All attempts at replication were successful.
Randomization	Samples were collected like pan-cancer real world study. This study didn't control the covariates.
Blinding	Throughout the data acquisition phase, the investigators maintained cognizance of the group allocation.

Reporting for specific materials, systems and methods

We require information from authors about some types of materials, experimental systems and methods used in many studies. Here, indicate whether each material, system or method listed is relevant to your study. If you are not sure if a list item applies to your research, read the appropriate section before selecting a response.

Materials & experimental systems

n/a	Involved in the study
☐	☒ Antibodies
☐	☒ Eukaryotic cell lines
☒	☐ Palaeontology and archaeology
☐	☒ Animals and other organisms
☒	☐ Clinical data
☒	☐ Dual use research of concern
☒	☐ Plants

Methods

n/a	Involved in the study
☒	☐ ChIP-seq
☒	☐ Flow cytometry
☒	☐ MRI-based neuroimaging

Antibodies

Antibodies used	Antibodies against CLDN18 (#ab222512, Abcam, dilution ratio 1:200); goat anti-rabbit IgG H&L (PV-9003, ZSGB-BIO Corp., dilution ratio 1:1000)
Validation	All antibodies were used based on the validation statements on the manufacturer's website, including the specie and applications.

Eukaryotic cell lines

Policy information about cell lines and Sex and Gender in Research

Cell line source(s)	Human GC cell lines MKN-1 and HGC-27 were obtained from Shanghai Bioleaf Biotech Co., Ltd., Shanghai, China.
Authentication	Authentication of these cell lines was conducted using Short Tandem Repeat analysis, and regular testing was carried out to ensure the absence of mycoplasma contamination.
Mycoplasma contamination	Authentication of these cell lines was conducted using Short Tandem Repeat analysis, and regular testing was carried out to ensure the absence of mycoplasma contamination.
Commonly misidentified lines (See ICLAC register)	No commonly misidentified cell lines were used.

Animals and other research organisms

Policy information about studies involving animals; ARRIVE guidelines recommended for reporting animal research, and Sex and Gender in Research

Laboratory animals	In accordance with the protocols for experimentation on animals (National Institutes of Health Publication No. 85-23, revised 1996), the animal experiments conducted were approved by the Institutional Animal Care and Use Committee of Zhejiang Chinese Medical University (The Ethics Committee stipulates that the xenograft and orthotopic tumor volume of mice should not exceed 2000 mm3 , and our experiments meet the ethical requirements.). The nude mice (male, 4 weeks old) were raised in the laboratory for a week before the experiment. Mice were fed in the Specific Pathogen Free (SPF) barrier center at the animal experimental center of Zhejiang Chinese Medical University, under standard conditions of temperature (25 ± 2 °C) and humility (50 ± 5%) in a 12 h light/12 h dark cycle with normal drink and food.
Wild animals	No wild animals were used in the study.
Reporting on sex	Due to the fact that ovarian metastasis from gastric cancer only occurs in female patients, our study included only 74 women, and the conclusions of this study are only applicable to female patients with ovarian metastasis from gastric cancer.
Field-collected samples	No field collected samples were used in the study.
Ethics oversight	The animal experiments were conducted with the approval of the Animal Ethical Committee at the institute of Zhejiang Chinese Medical University (202110-0682).

Note that full information on the approval of the study protocol must also be provided in the manuscript.
