# Supplementary figures and images for: Mutation characteristics and molecular evolution of ovarian metastasis from gastric cancer and potential biomarkers for paclitaxel treatment
Source: Nat Commun. 2024 May 4;15:3771. doi: 10.1038/s41467-024-48144-0 (PMC11069556; doi:10.1038/s41467-024-48144-0)

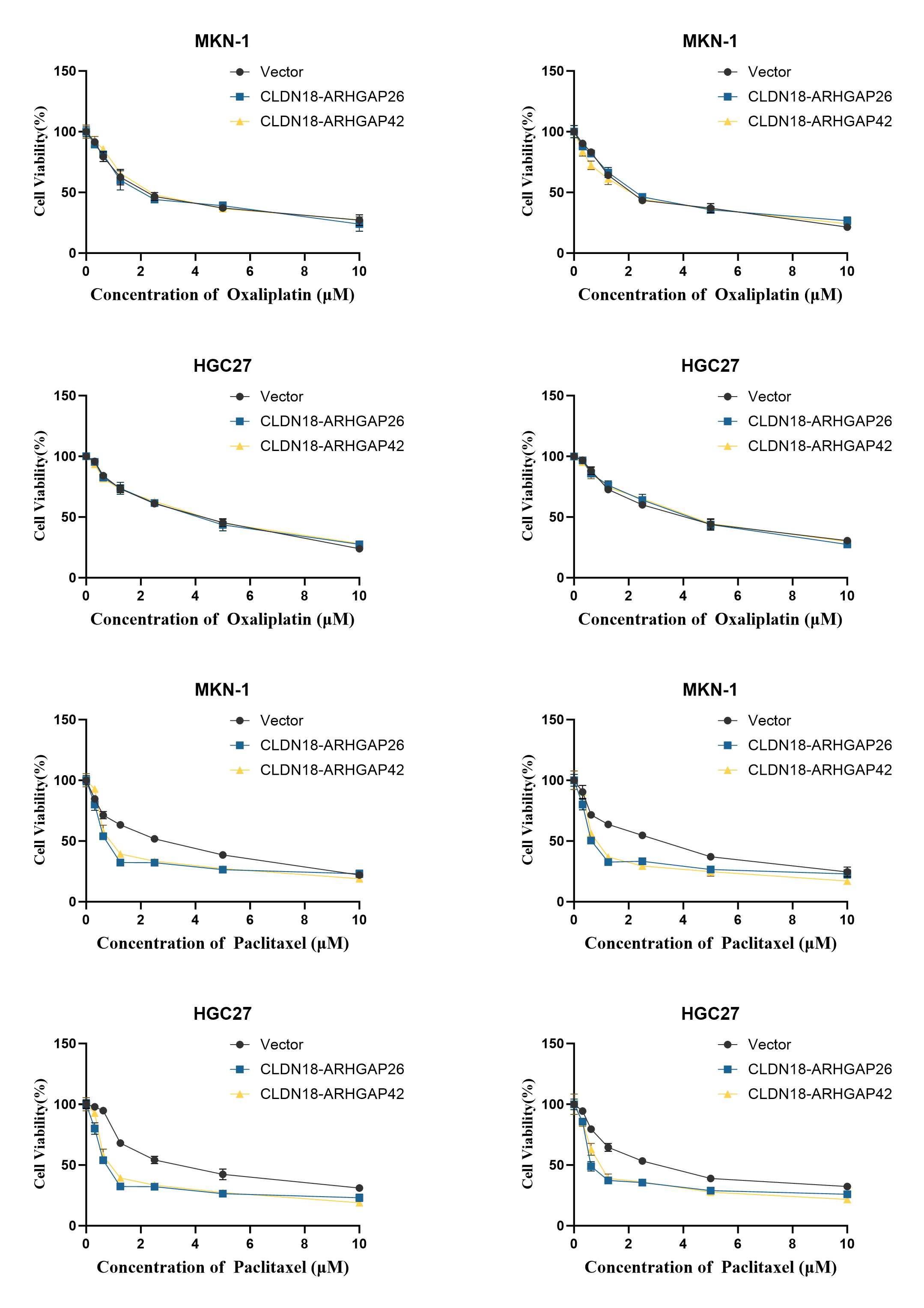

Supplement: Supplementary file 6 — Source Data [file 41467_2024_48144_MOESM6_ESM.zip › Separate Source Data files/Source data of Figure 6c.tif]

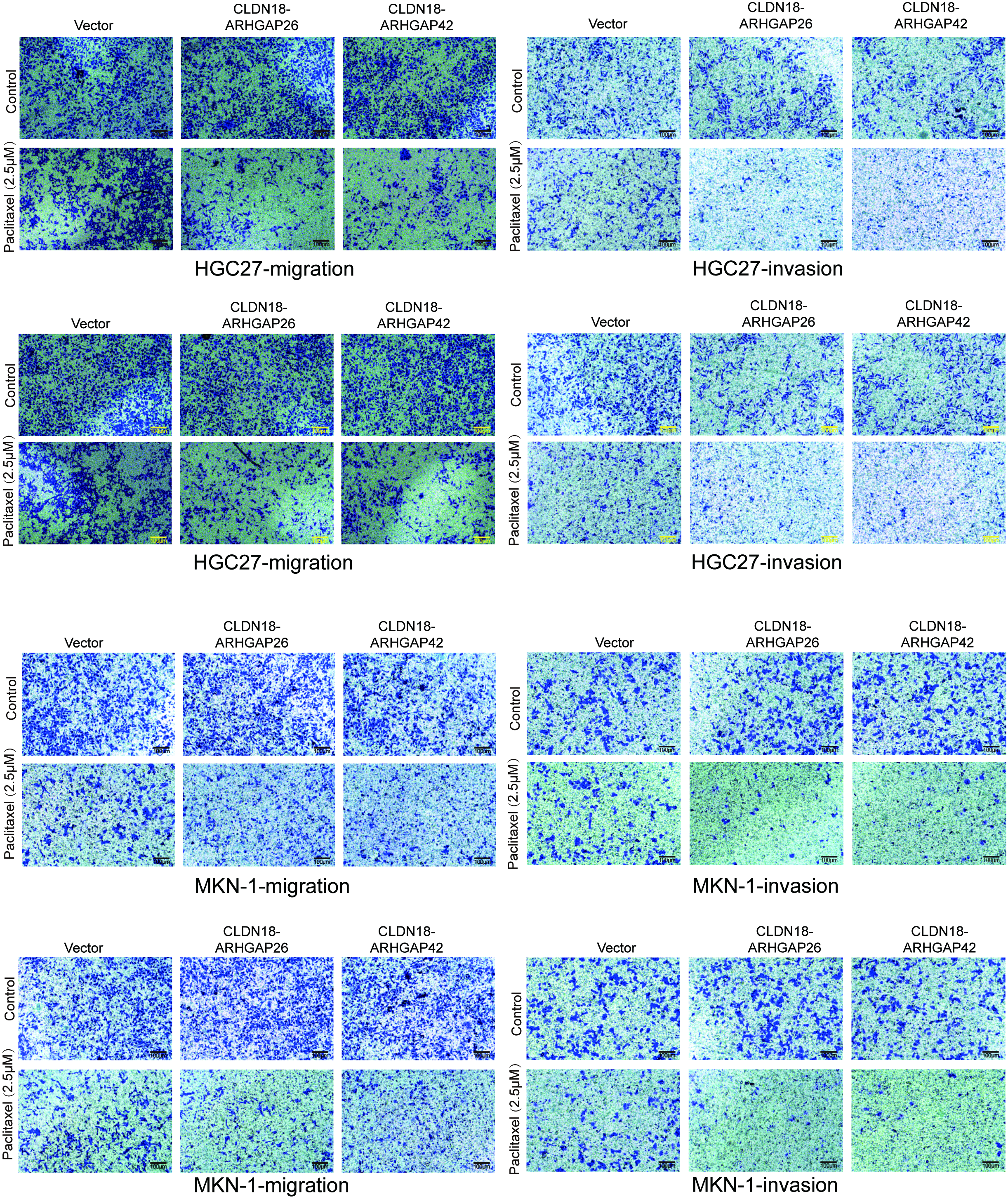

Supplement: Supplementary file 6 — Source Data [file 41467_2024_48144_MOESM6_ESM.zip › Separate Source Data files/Source data of Figure 6d.tif]
